# Supplementary material for: Whole Genome Sequencing and Comparative Analysis of the First Ehrlichia canis Isolate in China
Source: Microorganisms. 2024 Jan 8;12(1):125. doi: 10.3390/microorganisms12010125 (PMC10820421; doi:10.3390/microorganisms12010125)
Supplement: Supplementary file 1 [file microorganisms-12-00125-s001.zip › microorganisms-2797756-non-published.pdf]

map number	Functional Category	# of Gene	Gene #	KO #
map00010	Glycolysis / Gluconeogenesis	12	EC-YZGM001016	K00134
			EC-YZGM000178	K00161
			EC-YZGM000115	K00162
			EC-YZGM000462	K00382
			EC-YZGM000874	K00382
			EC-YZGM000067	K00627
			EC-YZGM000029	K00927
			EC-YZGM000065	K01623
			EC-YZGM000495	K01689
			EC-YZGM000595	K01803
			EC-YZGM000460	K01834
			EC-YZGM000328	K02446
map00020	Citrate cycle (TCA cycle)	16	EC-YZGM000590	K00024
			EC-YZGM000966	K00031
			EC-YZGM000178	K00161
			EC-YZGM000115	K00162
			EC-YZGM000745	K00164
			EC-YZGM000291	K00239
			EC-YZGM000292	K00240
			EC-YZGM000462	K00382
			EC-YZGM000874	K00382
			EC-YZGM000067	K00627
			EC-YZGM000931	K00658
			EC-YZGM000089	K01647
			EC-YZGM000342	K01679
			EC-YZGM000901	K01681
			EC-YZGM000864	K01902
			EC-YZGM000863	K01903
map00030	Pentose phosphate pathway	7	EC-YZGM000418	K00615
			EC-YZGM000525	K00616
			EC-YZGM000899	K00948
			EC-YZGM000065	K01623
			EC-YZGM000053	K01783
			EC-YZGM000587	K01808
			EC-YZGM000328	K02446
map00040	Pentose and glucuronate intercon	1	EC-YZGM000053	K01783
map00051	Fructose and mannose metabolisr	4	EC-YZGM000152	K00100
			EC-YZGM000065	K01623
			EC-YZGM000595	K01803
			EC-YZGM000328	K02446
map00061	Fatty acid biosynthesis	7	EC-YZGM000615	K00059
			EC-YZGM000729	K00208
			EC-YZGM000188	K00645
			EC-YZGM000404	K00648
			EC-YZGM000859	K02371
			EC-YZGM000939	K02372

			EC-YZGM000788	K09458
map00130	Ubiquinone and other terpenoid-c	6	EC-YZGM000586	K00568
			EC-YZGM000396	K03179
			EC-YZGM000397	K03179
			EC-YZGM000879	K03183
			EC-YZGM000001	K03185
			EC-YZGM000781	K06134
map00190	Oxidative phosphorylation	38	EC-YZGM000291	K00239
			EC-YZGM000292	K00240
			EC-YZGM000037	K00329
			EC-YZGM000303	K00329
			EC-YZGM000707	K00330
			EC-YZGM000708	K00331
			EC-YZGM000709	K00332
			EC-YZGM000562	K00333
			EC-YZGM000561	K00334
			EC-YZGM000498	K00335
			EC-YZGM000566	K00336
			EC-YZGM000565	K00337
			EC-YZGM000628	K00338
			EC-YZGM000508	K00339
			EC-YZGM000509	K00340
			EC-YZGM000510	K00341
			EC-YZGM000511	K00342
			EC-YZGM000140	K00343
			EC-YZGM000512	K00343
			EC-YZGM000143	K00356
			EC-YZGM000171	K00356
			EC-YZGM000471	K00411
			EC-YZGM000472	K00412
			EC-YZGM000473	K00413
			EC-YZGM000892	K01507
			EC-YZGM000947	K02108
			EC-YZGM000949	K02109
			EC-YZGM000950	K02109
			EC-YZGM000948	K02110
			EC-YZGM000098	K02111
			EC-YZGM000527	K02112
			EC-YZGM000097	K02113
			EC-YZGM000526	K02114
			EC-YZGM000600	K02115
			EC-YZGM000883	K02274
			EC-YZGM000882	K02275
			EC-YZGM000010	K02276
			EC-YZGM000884	K02301
map00195	Photosynthesis	9	EC-YZGM000947	K02108
			EC-YZGM000949	K02109

			EC-YZGM000950	K02109
			EC-YZGM000948	K02110
			EC-YZGM000098	K02111
			EC-YZGM000527	K02112
			EC-YZGM000097	K02113
			EC-YZGM000526	K02114
			EC-YZGM000600	K02115
map00230	Purine metabolism	34	EC-YZGM000185	K00088
			EC-YZGM000413	K00525
			EC-YZGM000682	K00526
			EC-YZGM000940	K00602
			EC-YZGM000106	K00764
			EC-YZGM000386	K00939
			EC-YZGM000969	K00940
			EC-YZGM000299	K00942
			EC-YZGM000899	K00948
			EC-YZGM000653	K00962
			EC-YZGM000625	K01129
			EC-YZGM000123	K01588
			EC-YZGM000903	K01589
			EC-YZGM000764	K01756
			EC-YZGM000264	K01923
			EC-YZGM000315	K01933
			EC-YZGM000415	K01939
			EC-YZGM000886	K01945
			EC-YZGM000088	K01951
			EC-YZGM000323	K01952
			EC-YZGM000330	K01952
			EC-YZGM000052	K02335
			EC-YZGM000822	K02337
			EC-YZGM000888	K02338
			EC-YZGM000091	K02340
			EC-YZGM000722	K02341
			EC-YZGM000477	K02342
			EC-YZGM001019	K02343
			EC-YZGM000389	K03040
			EC-YZGM000841	K03043
			EC-YZGM000840	K03046
			EC-YZGM000717	K03060
			EC-YZGM000712	K03787
			EC-YZGM000338	K11175
map00240	Pyrimidine metabolism	33	EC-YZGM000831	K00226
			EC-YZGM000597	K00384
			EC-YZGM000660	K00384
			EC-YZGM000413	K00525
			EC-YZGM000682	K00526
			EC-YZGM000569	K00609

			EC-YZGM000962	K00762
			EC-YZGM000969	K00940
			EC-YZGM000189	K00943
			EC-YZGM000362	K00945
			EC-YZGM000653	K00962
			EC-YZGM000340	K01465
			EC-YZGM000406	K01485
			EC-YZGM000270	K01494
			EC-YZGM000456	K01520
			EC-YZGM000713	K01591
			EC-YZGM000132	K01937
			EC-YZGM000344	K01955
			EC-YZGM000458	K01956
			EC-YZGM000052	K02335
			EC-YZGM000822	K02337
			EC-YZGM000888	K02338
			EC-YZGM000091	K02340
			EC-YZGM000722	K02341
			EC-YZGM000477	K02342
			EC-YZGM001019	K02343
			EC-YZGM000389	K03040
			EC-YZGM000841	K03043
			EC-YZGM000840	K03046
			EC-YZGM000717	K03060
			EC-YZGM000294	K03465
			EC-YZGM000712	K03787
			EC-YZGM000212	K09903
map00250	Alanine, aspartate and glutamate	13	EC-YZGM000689	K00260
			EC-YZGM000569	K00609
			EC-YZGM000106	K00764
			EC-YZGM000658	K00812
			EC-YZGM000829	K01755
			EC-YZGM000764	K01756
			EC-YZGM000059	K01915
			EC-YZGM000868	K01915
			EC-YZGM000415	K01939
			EC-YZGM000622	K01940
			EC-YZGM000344	K01955
			EC-YZGM000458	K01956
			EC-YZGM000614	K13821
map00260	Glycine, serine and threonine met	7	EC-YZGM001021	K00133
			EC-YZGM000462	K00382
			EC-YZGM000874	K00382
			EC-YZGM000287	K00600
			EC-YZGM000061	K00643
			EC-YZGM000881	K00928
			EC-YZGM000701	K00998

map00270	Cysteine and methionine metabol	4	EC-YZGM001021	K00133
			EC-YZGM001022	K00789
			EC-YZGM000658	K00812
			EC-YZGM000881	K00928
map00280	Valine, leucine and isoleucine deg	4	EC-YZGM000462	K00382
			EC-YZGM000874	K00382
			EC-YZGM000444	K01965
			EC-YZGM000548	K01966
map00290	Valine, leucine and isoleucine bios	5	EC-YZGM000178	K00161
			EC-YZGM000115	K00162
			EC-YZGM000715	K01869
			EC-YZGM000489	K01870
			EC-YZGM000104	K01873
map00300	Lysine biosynthesis	11	EC-YZGM001021	K00133
			EC-YZGM000893	K00145
			EC-YZGM000399	K00215
			EC-YZGM000033	K00674
			EC-YZGM000792	K00821
			EC-YZGM000881	K00928
			EC-YZGM000542	K00930
			EC-YZGM000110	K01439
			EC-YZGM000442	K01586
			EC-YZGM000742	K01714
			EC-YZGM000027	K01778
map00310	Lysine degradation	3	EC-YZGM000745	K00164
			EC-YZGM000931	K00658
			EC-YZGM000656	K01423
map00330	Arginine and proline metabolism	14	EC-YZGM000893	K00145
			EC-YZGM000689	K00260
			EC-YZGM001018	K00286
			EC-YZGM000050	K00611
			EC-YZGM000619	K00620
			EC-YZGM000658	K00812
			EC-YZGM000792	K00821
			EC-YZGM000542	K00930
			EC-YZGM000406	K01485
			EC-YZGM000829	K01755
			EC-YZGM000059	K01915
			EC-YZGM000868	K01915
			EC-YZGM000622	K01940
			EC-YZGM000614	K13821
map00340	Histidine metabolism	2	EC-YZGM000398	K00492
			EC-YZGM000672	K00599
map00350	Tyrosine metabolism	3	EC-YZGM000672	K00599
			EC-YZGM000810	K00680
			EC-YZGM000658	K00812
map00360	Phenylalanine metabolism	1	EC-YZGM000658	K00812

map00361	Chlorocyclohexane and chloroben	1	EC-YZGM000398	K00492
map00362	Benzoate degradation	2	EC-YZGM000336	K00449
			EC-YZGM000810	K00680
map00363	Bisphenol degradation	2	EC-YZGM000152	K00100
			EC-YZGM000398	K00492
map00380	Tryptophan metabolism	2	EC-YZGM000745	K00164
			EC-YZGM000128	K01867
map00400	Phenylalanine, tyrosine and tryptc	2	EC-YZGM000821	K00800
			EC-YZGM000658	K00812
map00401	Novobiocin biosynthesis	1	EC-YZGM000658	K00812
map00410	beta-Alanine metabolism	1	EC-YZGM000731	K01578
map00450	Selenocompound metabolism	3	EC-YZGM000597	K00384
			EC-YZGM000660	K00384
			EC-YZGM000880	K01874
map00460	Cyanoamino acid metabolism	1	EC-YZGM000287	K00600
map00480	Glutathione metabolism	5	EC-YZGM000966	K00031
			EC-YZGM000760	K00799
			EC-YZGM000337	K01255
			EC-YZGM000090	K01919
			EC-YZGM000310	K01920
map00500	Starch and sucrose metabolism	1	EC-YZGM000639	K00975
map00520	Amino sugar and nucleotide sugar	1	EC-YZGM000639	K00975
map00521	Streptomycin biosynthesis	1	EC-YZGM000693	K01092
map00550	Peptidoglycan biosynthesis	1	EC-YZGM000933	K07258
map00561	Glycerolipid metabolism	3	EC-YZGM000045	K00655
			EC-YZGM000403	K03621
			EC-YZGM000008	K08591
map00562	Inositol phosphate metabolism	2	EC-YZGM000693	K01092
			EC-YZGM000595	K01803
map00564	Glycerophospholipid metabolism	10	EC-YZGM000313	K00057
			EC-YZGM000045	K00655
			EC-YZGM000214	K00981
			EC-YZGM000942	K00995
			EC-YZGM000701	K00998
			EC-YZGM000809	K01095
			EC-YZGM000696	K01613
			EC-YZGM000700	K01613
			EC-YZGM000403	K03621
			EC-YZGM000008	K08591
map00591	Linoleic acid metabolism	1	EC-YZGM000152	K00100
map00620	Pyruvate metabolism	8	EC-YZGM000590	K00024
			EC-YZGM000136	K00029
			EC-YZGM000178	K00161
			EC-YZGM000115	K00162
			EC-YZGM000462	K00382
			EC-YZGM000874	K00382
			EC-YZGM000067	K00627

			EC-YZGM000305	K01006
map00623	Toluene degradation	3	EC-YZGM000291	K00239
			EC-YZGM000292	K00240
			EC-YZGM000398	K00492
map00624	Polycyclic aromatic hydrocarbon d	3	EC-YZGM000336	K00449
			EC-YZGM000398	K00492
			EC-YZGM000672	K00599
map00625	Chloroalkane and chloroalkene de	1	EC-YZGM000152	K00100
map00626	Naphthalene degradation	2	EC-YZGM000398	K00492
			EC-YZGM000810	K00680
map00627	Aminobenzoate degradation	2	EC-YZGM000398	K00492
			EC-YZGM000810	K00680
map00630	Glyoxylate and dicarboxylate meta	6	EC-YZGM000590	K00024
			EC-YZGM000307	K01091
			EC-YZGM000089	K01647
			EC-YZGM000901	K01681
			EC-YZGM000444	K01965
			EC-YZGM000548	K01966
map00640	Propanoate metabolism	5	EC-YZGM000731	K01578
			EC-YZGM000864	K01902
			EC-YZGM000863	K01903
			EC-YZGM000444	K01965
			EC-YZGM000548	K01966
map00642	Ethylbenzene degradation	1	EC-YZGM000810	K00680
map00650	Butanoate metabolism	5	EC-YZGM000152	K00100
			EC-YZGM000178	K00161
			EC-YZGM000115	K00162
			EC-YZGM000291	K00239
			EC-YZGM000292	K00240
map00660	C5-Branched dibasic acid metabol	2	EC-YZGM000864	K01902
			EC-YZGM000863	K01903
map00670	One carbon pool by folate	7	EC-YZGM000287	K00600
			EC-YZGM000940	K00602
			EC-YZGM000801	K00604
			EC-YZGM000300	K01491
			EC-YZGM000492	K01934
			EC-YZGM000294	K03465
			EC-YZGM000338	K11175
map00680	Methane metabolism	4	EC-YZGM000590	K00024
			EC-YZGM000287	K00600
			EC-YZGM000495	K01689
			EC-YZGM000460	K01834
map00710	Carbon fixation in photosynthetic	11	EC-YZGM000590	K00024
			EC-YZGM000136	K00029
			EC-YZGM000418	K00615
			EC-YZGM000658	K00812
			EC-YZGM000029	K00927

			EC-YZGM000305	K01006
			EC-YZGM000065	K01623
			EC-YZGM000053	K01783
			EC-YZGM000595	K01803
			EC-YZGM000587	K01808
			EC-YZGM000328	K02446
map00720	Carbon fixation pathways in proka	11	EC-YZGM000590	K00024
			EC-YZGM000966	K00031
			EC-YZGM000291	K00239
			EC-YZGM000292	K00240
			EC-YZGM000300	K01491
			EC-YZGM000342	K01679
			EC-YZGM000901	K01681
			EC-YZGM000864	K01902
			EC-YZGM000863	K01903
			EC-YZGM000444	K01965
			EC-YZGM000548	K01966
map00730	Thiamine metabolism	7	EC-YZGM000798	K00788
			EC-YZGM000816	K00941
			EC-YZGM000478	K00946
			EC-YZGM000961	K03148
			EC-YZGM000409	K03153
			EC-YZGM000577	K04487
			EC-YZGM000578	K04487
map00740	Riboflavin metabolism	6	EC-YZGM000196	K00793
			EC-YZGM000704	K00794
			EC-YZGM000024	K01497
			EC-YZGM000100	K02858
			EC-YZGM000130	K11752
			EC-YZGM000927	K11753
map00750	Vitamin B6 metabolism	2	EC-YZGM000826	K00275
			EC-YZGM000724	K03474
map00760	Nicotinate and nicotinamide meta	6	EC-YZGM000007	K00767
			EC-YZGM000186	K00858
			EC-YZGM000725	K00969
			EC-YZGM000738	K01950
			EC-YZGM000016	K03517
			EC-YZGM000712	K03787
map00770	Pantothenate and CoA biosynthes	6	EC-YZGM000720	K00859
			EC-YZGM000662	K00954
			EC-YZGM000665	K00997
			EC-YZGM000035	K01598
			EC-YZGM000341	K01922
			EC-YZGM000832	K03525
map00780	Biotin metabolism	6	EC-YZGM000839	K00652
			EC-YZGM000613	K00833
			EC-YZGM000324	K01012

			EC-YZGM000656	K01423
			EC-YZGM000964	K01935
			EC-YZGM000761	K03524
map00785	Lipoic acid metabolism	2	EC-YZGM000446	K03644
			EC-YZGM000339	K03801
map00790	Folate biosynthesis	5	EC-YZGM000346	K00796
			EC-YZGM000321	K00950
			EC-YZGM000599	K01495
			EC-YZGM000591	K01633
			EC-YZGM000637	K11754
map00860	Porphyrin and chlorophyll metabo	10	EC-YZGM000540	K00228
			EC-YZGM000061	K00643
			EC-YZGM000011	K01599
			EC-YZGM000737	K01698
			EC-YZGM000437	K01719
			EC-YZGM000636	K01749
			EC-YZGM000355	K01772
			EC-YZGM000166	K01885
			EC-YZGM000554	K01885
			EC-YZGM000884	K02301
map00900	Terpenoid backbone biosynthesis	8	EC-YZGM000513	K00099
			EC-YZGM000411	K00795
			EC-YZGM000675	K00919
			EC-YZGM000120	K00991
			EC-YZGM000119	K01770
			EC-YZGM000057	K02523
			EC-YZGM000515	K03526
			EC-YZGM000457	K03527
map00903	Limonene and pinene degradatior	2	EC-YZGM000398	K00492
			EC-YZGM000810	K00680
map00908	Zeatin biosynthesis	1	EC-YZGM000529	K00791
map00910	Nitrogen metabolism	3	EC-YZGM000689	K00260
			EC-YZGM000059	K01915
			EC-YZGM000868	K01915
map00950	Isoquinoline alkaloid biosynthesis	1	EC-YZGM000658	K00812
map00960	Tropane, piperidine and pyridine a	1	EC-YZGM000658	K00812
map00970	Aminoacyl-tRNA biosynthesis	25	EC-YZGM000801	K00604
			EC-YZGM000060	K01866
			EC-YZGM000128	K01867
			EC-YZGM001011	K01868
			EC-YZGM000715	K01869
			EC-YZGM000489	K01870
			EC-YZGM000865	K01872
			EC-YZGM000104	K01873
			EC-YZGM000880	K01874
			EC-YZGM000532	K01875
			EC-YZGM000308	K01876

			EC-YZGM000003	K01878
			EC-YZGM000004	K01879
			EC-YZGM000664	K01881
			EC-YZGM000687	K01883
			EC-YZGM000166	K01885
			EC-YZGM000554	K01885
			EC-YZGM000485	K01887
			EC-YZGM000153	K01889
			EC-YZGM000391	K01890
			EC-YZGM000265	K01892
			EC-YZGM000638	K02433
			EC-YZGM000730	K02434
			EC-YZGM000900	K02435
			EC-YZGM000575	K04566
map00980	Metabolism of xenobiotics by cytc	1	EC-YZGM000760	K00799
map00982	Drug metabolism - cytochrome P4	1	EC-YZGM000760	K00799
map00983	Drug metabolism - other enzymes	2	EC-YZGM000185	K00088
			EC-YZGM000088	K01951
map01040	Biosynthesis of unsaturated fatty a	1	EC-YZGM000615	K00059
map01051	Biosynthesis of ansamycins	1	EC-YZGM000418	K00615
map02010	ABC transporters	5	EC-YZGM000758	K02010
			EC-YZGM000400	K02036
			EC-YZGM000269	K02193
			EC-YZGM000135	K09810
			EC-YZGM000753	K09817
map02020	Two-component system	4	EC-YZGM000059	K01915
			EC-YZGM000868	K01915
			EC-YZGM000791	K07716
			EC-YZGM000273	K13598
map03008	Ribosome biogenesis in eukaryote	1	EC-YZGM000921	K03685
map03018	RNA degradation	4	EC-YZGM000653	K00962
			EC-YZGM000968	K00970
			EC-YZGM000495	K01689
			EC-YZGM000424	K08300
map03020	RNA polymerase	4	EC-YZGM000389	K03040
			EC-YZGM000841	K03043
			EC-YZGM000840	K03046
			EC-YZGM000717	K03060
map03030	DNA replication	12	EC-YZGM000277	K01972
			EC-YZGM000408	K02314
			EC-YZGM000678	K02316
			EC-YZGM000052	K02335
			EC-YZGM000822	K02337
			EC-YZGM000888	K02338
			EC-YZGM000091	K02340
			EC-YZGM000722	K02341
			EC-YZGM000477	K02342

			EC-YZGM001019	K02343
			EC-YZGM000210	K03469
			EC-YZGM000837	K03470
map03060	Protein export	2	EC-YZGM000627	K03100
			EC-YZGM000926	K03101
map03410	Base excision repair	6	EC-YZGM000618	K01142
			EC-YZGM000277	K01972
			EC-YZGM000052	K02335
			EC-YZGM000222	K03652
			EC-YZGM000967	K07462
			EC-YZGM000767	K10773
map03420	Nucleotide excision repair	4	EC-YZGM000277	K01972
			EC-YZGM000052	K02335
			EC-YZGM000769	K03657
			EC-YZGM000204	K03723
map03430	Mismatch repair	11	EC-YZGM000277	K01972
			EC-YZGM000822	K02337
			EC-YZGM000888	K02338
			EC-YZGM000091	K02340
			EC-YZGM000722	K02341
			EC-YZGM000477	K02342
			EC-YZGM001019	K02343
			EC-YZGM000031	K03601
			EC-YZGM000172	K03602
			EC-YZGM000769	K03657
			EC-YZGM000967	K07462
map03440	Homologous recombination	11	EC-YZGM000009	K01159
			EC-YZGM000052	K02335
			EC-YZGM000822	K02337
			EC-YZGM000888	K02338
			EC-YZGM000091	K02340
			EC-YZGM000722	K02341
			EC-YZGM000477	K02342
			EC-YZGM001019	K02343
			EC-YZGM000036	K03655
			EC-YZGM000440	K04066
			EC-YZGM000967	K07462
map04070	Phosphatidylinositol signaling syst	2	EC-YZGM000214	K00981
			EC-YZGM000693	K01092
map04112	Cell cycle - Caulobacter	5	EC-YZGM000804	K01338
			EC-YZGM000806	K01358
			EC-YZGM000408	K02314
			EC-YZGM000791	K07716
			EC-YZGM000936	K11749
map04122	Sulfur relay system	4	EC-YZGM000780	K00566
			EC-YZGM000961	K03148
			EC-YZGM000577	K04487

			EC-YZGM000578	K04487
map04146	Peroxisome	3	EC-YZGM000966	K00031
			EC-YZGM000731	K01578
			EC-YZGM000448	K04564
map04260	Cardiac muscle contraction	3	EC-YZGM000471	K00411
			EC-YZGM000472	K00412
			EC-YZGM000473	K00413
map04724	Glutamatergic synapse	2	EC-YZGM000059	K01915
			EC-YZGM000868	K01915
map05010	Alzheimer's disease	4	EC-YZGM001016	K00134
			EC-YZGM000471	K00411
			EC-YZGM000472	K00412
			EC-YZGM000473	K00413
map05012	Parkinson's disease	3	EC-YZGM000471	K00411
			EC-YZGM000472	K00412
			EC-YZGM000473	K00413
map05016	Huntington's disease	4	EC-YZGM000471	K00411
			EC-YZGM000472	K00412
			EC-YZGM000473	K00413
			EC-YZGM000448	K04564
map05211	Renal cell carcinoma	1	EC-YZGM000342	K01679

Gene
GAPDH, gapA
PDHA, pdhA
PDHB, pdhB
DLD, lpd, pdhD
DLD, lpd, pdhD
DLAT, aceF, pdhC
PGK, pgk
ALDO, fbaB
ENO, eno
TPI, tpiA
PGAM, gpm
glpX
mdh
IDH1, IDH2, icd
PDHA, pdhA
PDHB, pdhB
OGDH, sucA
sdhA
sdhB
DLD, lpd, pdhD
DLD, lpd, pdhD
DLAT, aceF, pdhC
DLST, sucB
CS, gltA
E4.2.1.2B, fumC
ACO, acnA
sucD
sucC
E2.2.1.1, tktA, tktB
E2.2.1.2, talA, talB
PRPS, prsA
ALDO, fbaB
rpe, RPE
E5.3.1.6B, rpiB
glpX
rpe, RPE
E1.1.1.-
ALDO, fbaB
TPI, tpiA
glpX
fabG
fabI
fabD
fabH
fabK
fabZ

fabF
ubiG
ubiA
ubiA
ubiE
ubiH
COQ7
sdhA
sdhB
E1.6.5.3
E1.6.5.3
nuoA
nuoB
nuoC
nuoD
nuoE
nuoF
nuoG
nuoH
nuoI
nuoJ
nuoK
nuoL
nuoM
nuoN
nuoN
E1.6.99.3
E1.6.99.3
RIP1, UQCRFS1, petA
CYTB, petB
CYT1, CYC1, petC
E3.6.1.1, ppa
ATPF0A, atpB
ATPF0B, atpF
ATPF0B, atpF
ATPF0C, atpE
ATPF1A, atpA
ATPF1B, atpD
ATPF1D, atpH
ATPF1E, atpC
ATPF1G, atpG
coxA
coxB
coxC
cyoE
ATPF0A, atpB
ATPF0B, atpF

ATPF0B, atpF
ATPF0C, atpE
ATPF1A, atpA
ATPF1B, atpD
ATPF1D, atpH
ATPF1E, atpC
ATPF1G, atpG
E1.1.1.205, guaB
E1.17.4.1A, nrdA, nrdE
E1.17.4.1B, nrdB, nrdF
purH
E2.4.2.14, purF
E2.7.4.3, adk
E2.7.4.6, ndk
E2.7.4.8, gmk
PRPS, prsA
pnp, PNPT1
dgt
purE
purK
E4.3.2.2, purB
purC
purM
E6.3.4.4, purA
purD
E6.3.5.2, guaA
E6.3.5.3, purL
E6.3.5.3, purL
DPO1, polA
DPO3A1, dnaE
DPO3B, dnaN
DPO3D1, holA
DPO3D2, holB
DPO3E, dnaQ
DPO3G, dnaX
rpoA
rpoB
rpoC
rpoZ
surE
purN
pyrD
E1.8.1.9, trxB
E1.8.1.9, trxB
E1.17.4.1A, nrdA, nrdE
E1.17.4.1B, nrdB, nrdF
pyrB, PYR2

pyrE
E2.7.4.6, ndk
E2.7.4.9, tmk
cmk
pnp, PNPT1
URA4, pyrC
E3.5.4.1, codA
E3.5.4.13, dcd
E3.6.1.23, dut
pyrF
E6.3.4.2, pyrG
carB, CPA2
carA, CPA1
DPO1, polA
DPO3A1, dnaE
DPO3B, dnaN
DPO3D1, holA
DPO3D2, holB
DPO3E, dnaQ
DPO3G, dnaX
rpoA
rpoB
rpoC
rpoZ
E2.1.1.148, thyX, thy1
surE
pyrH
E1.4.1.2
pyrB, PYR2
E2.4.2.14, purF
E2.6.1.1A, aspB
argH, ASL
E4.3.2.2, purB
E6.3.1.2, glnA
E6.3.1.2, glnA
E6.3.4.4, purA
E6.3.4.5, argG
carB, CPA2
carA, CPA1
putA
E1.2.1.11, asd
DLD, lpd, pdhD
DLD, lpd, pdhD
E2.1.2.1, glyA
E2.3.1.37, ALAS
E2.7.2.4, lysC
E2.7.8.8, pssA

E1.2.1.11, asd
E2.5.1.6, metK
E2.6.1.1A, aspB
E2.7.2.4, lysC
DLD, lpd, pdhD
DLD, lpd, pdhD
PCCA, pccA
PCCB, pccB
PDHA, pdhA
PDHB, pdhB
LARS, leuS
IARS, ileS
VARS, valS
E1.2.1.11, asd
argC
dapB
dapD
argD
E2.7.2.4, lysC
argB
dapE
lysA
dapA
dapF
OGDH, sucA
DLST, sucB
E3.4.-.-
argC
E1.4.1.2
E1.5.1.2, proC
OTC, argF, argI
argJ
E2.6.1.1A, aspB
argD
argB
E3.5.4.1, codA
argH, ASL
E6.3.1.2, glnA
E6.3.1.2, glnA
E6.3.4.5, argG
putA
E1.14.13.-
E2.1.1.-
E2.1.1.-
E2.3.1.-
E2.6.1.1A, aspB
E2.6.1.1A, aspB

E1.14.13.-
pcaH
E2.3.1.-
E1.1.1.-
E1.14.13.-
OGDH, sucA
WARS, trpS
aroA
E2.6.1.1A, aspB
E2.6.1.1A, aspB
E4.1.1.9, MLYCD
E1.8.1.9, trxB
E1.8.1.9, trxB
MARS, metG
E2.1.2.1, glyA
IDH1, IDH2, icd
E2.5.1.18, gst
CARP, pepA
gshA
E6.3.2.3, gshB
glgC
glgC
E3.1.3.25, IMPA, suhB
dacC, dacA, dacD
plsC
plsX
plsY
E3.1.3.25, IMPA, suhB
TPI, tpiA
gpsA
plsC
E2.7.7.41, CDS1, cdsA
E2.7.8.5, pgsA
E2.7.8.8, pssA
pgpA
E4.1.1.65, psd
E4.1.1.65, psd
plsX
plsY
E1.1.1.-
mdh
E1.1.1.40, maeB
PDHA, pdhA
PDHB, pdhB
DLD, lpd, pdhD
DLD, lpd, pdhD
DLAT, aceF, pdhC

ppdK
sdhA
sdhB
E1.14.13.-
pcaH
E1.14.13.-
E2.1.1.-
E1.1.1.-
E1.14.13.-
E2.3.1.-
E1.14.13.-
E2.3.1.-
mdh
E3.1.3.18, gph
CS, gltA
ACO, acnA
PCCA, pccA
PCCB, pccB
E4.1.1.9, MLYCD
sucD
sucC
PCCA, pccA
PCCB, pccB
E2.3.1.-
E1.1.1.-
PDHA, pdhA
PDHB, pdhB
sdhA
sdhB
sucD
sucC
E2.1.2.1, glyA
purH
MTFMT, fmt
folD
E6.3.3.2
E2.1.1.148, thyX, thy1
purN
mdh
E2.1.2.1, glyA
ENO, eno
PGAM, gpm
mdh
E1.1.1.40, maeB
E2.2.1.1, tktA, tktB
E2.6.1.1A, aspB
PGK, pgk

ppdK
ALDO, fbaB
rpe, RPE
TPI, tpiA
E5.3.1.6B, rpiB
glpX
mdh
IDH1, IDH2, icd
sdhA
sdhB
folD
E4.2.1.2B, fumC
ACO, acnA
sucD
sucC
PCCA, pccA
PCCB, pccB
thiE
thiD
thiL
thiF
thiO
iscS, NFS1
iscS, NFS1
ribE, RIB5
ribH, RIB4
ribA, RIB1
ribB, RIB3
ribD
ribF
pdxH, PNPO
pdxJ
E2.4.2.19, nadC
E2.7.1.23
nadD
E6.3.5.1, NADSYN1, QNS1, nadE
nadA
surE
coaE
E2.7.7.3A, coaD, kdtB
acpS
PPCDC, coaC
PPCS, coaB
coaX
bioF
E2.6.1.62, bioA
E2.8.1.6, bioB

E3.4.-.-
bioD
birA
lipA
lipB
folP
folK
E3.5.4.16, folE
folB
folC
hemF, CPOX
E2.3.1.37, ALAS
hemE, UROD
hemB, ALAD
hemD, UROS
hemC, HMBS
hemH, FECH
EARS, gltX
EARS, gltX
cyoE
dxr
ispA
ispE
ispD
ispF
ispB
E1.17.7.1, gcpE, ispG
E1.17.1.2, lytB, ispH
E1.14.13.-
E2.3.1.-
miaA, TRIT1
E1.4.1.2
E6.3.1.2, glnA
E6.3.1.2, glnA
E2.6.1.1A, aspB
E2.6.1.1A, aspB
MTFMT, fmt
YARS, tyrS
WARS, trpS
TARS, thrS
LARS, leuS
IARS, ileS
AARS, alaS
VARS, valS
MARS, metG
SARS, serS
DARS, aspS

glyQ
glyS
PARS, proS
CARS, cysS
EARS, gltX
EARS, gltX
RARS, argS
FARSA, pheS
FARSB, pheT
HARS, hisS
gatA
gatB
gatC
lysK
E2.5.1.18, gst
E2.5.1.18, gst
E1.1.1.205, guaB
E6.3.5.2, guaA
fabG
E2.2.1.1, tktA, tktB
afuC, fbpC
pstB
ccmA
ABC.LPT.A, lolD
znuC
E6.3.1.2, glnA
E6.3.1.2, glnA
pleC
ntrY
rnc, DROSHA, RNT1
pnp, PNPT1
pcnB
ENO, eno
rne
rpoA
rpoB
rpoC
rpoZ
E6.5.1.2, ligA, ligB
dnaB
dnaG
DPO1, polA
DPO3A1, dnaE
DPO3B, dnaN
DPO3D1, holA
DPO3D2, holB
DPO3E, dnaQ

DPO3G, dnaX
E3.1.26.4A, RNASEH1, rnhA
rnhB
lepB
lspA
E3.1.11.2, xthA
E6.5.1.2, ligA, ligB
DPO1, polA
MPG
recJ
NTH
E6.5.1.2, ligA, ligB
DPO1, polA
uvrD, pcrA
mfd
E6.5.1.2, ligA, ligB
DPO3A1, dnaE
DPO3B, dnaN
DPO3D1, holA
DPO3D2, holB
DPO3E, dnaQ
DPO3G, dnaX
xseA
xseB
uvrD, pcrA
recJ
ruvC
DPO1, polA
DPO3A1, dnaE
DPO3B, dnaN
DPO3D1, holA
DPO3D2, holB
DPO3E, dnaQ
DPO3G, dnaX
recG
priA
recJ
E2.7.7.41, CDS1, cdsA
E3.1.3.25, IMPA, suhB
lon
clpP, CLPP
dnaB
pleC
rseP
mnmA, trmU, TRMU
thiF
iscS, NFS1

iscS, NFS1
IDH1, IDH2, icd
E4.1.1.9, MLYCD
E1.15.1.1A, sodA, sodB, SOD2
RIP1, UQCRFS1, petA
CYTB, petB
CYT1, CYC1, petC
E6.3.1.2, glnA
E6.3.1.2, glnA
GAPDH, gapA
RIP1, UQCRFS1, petA
CYTB, petB
CYT1, CYC1, petC
RIP1, UQCRFS1, petA
CYTB, petB
CYT1, CYC1, petC
RIP1, UQCRFS1, petA
CYTB, petB
CYT1, CYC1, petC
E1.15.1.1A, sodA, sodB, SOD2
E4.2.1.2B, fumC

KO defination

glyceraldehyde 3-phosphate dehydrogenase
pyruvate dehydrogenase E1 component subunit alpha
pyruvate dehydrogenase E1 component subunit beta
dihydrolipoamide dehydrogenase
dihydrolipoamide dehydrogenase
pyruvate dehydrogenase E2 component (dihydrolipoamide acetyltransferase)
phosphoglycerate kinase
fructose-bisphosphate aldolase, class I
enolase
triosephosphate isomerase (TIM)
phosphoglycerate mutase
fructose-1,6-bisphosphatase II
malate dehydrogenase
isocitrate dehydrogenase
pyruvate dehydrogenase E1 component subunit alpha
pyruvate dehydrogenase E1 component subunit beta
2-oxoglutarate dehydrogenase E1 component
succinate dehydrogenase flavoprotein subunit
succinate dehydrogenase iron-sulfur protein
dihydrolipoamide dehydrogenase
dihydrolipoamide dehydrogenase
pyruvate dehydrogenase E2 component (dihydrolipoamide acetyltransferase)
2-oxoglutarate dehydrogenase E2 component (dihydrolipoamide succinyltransferase)
citrate synthase
fumarate hydratase, class II
aconitate hydratase 1
succinyl-CoA synthetase alpha subunit
succinyl-CoA synthetase beta subunit
transketolase
transaldolase
ribose-phosphate pyrophosphokinase
fructose-bisphosphate aldolase, class I
ribulose-phosphate 3-epimerase
ribose 5-phosphate isomerase B
fructose-1,6-bisphosphatase II
ribulose-phosphate 3-epimerase
--
fructose-bisphosphate aldolase, class I
triosephosphate isomerase (TIM)
fructose-1,6-bisphosphatase II
3-oxoacyl-[acyl-carrier protein] reductase
enoyl-[acyl-carrier protein] reductase I
[acyl-carrier-protein] S-malonyltransferase
3-oxoacyl-[acyl-carrier-protein] synthase III
enoyl-[acyl carrier protein] reductase II
3R-hydroxymyristoyl ACP dehydrase

3-oxoacyl-[acyl-carrier-protein] synthase II
3-demethylubiquinone-9 3-methyltransferase
4-hydroxybenzoate octaprenyltransferase
4-hydroxybenzoate octaprenyltransferase
ubiquinone/menaquinone biosynthesis methyltransferase
2-octaprenyl-6-methoxyphenol hydroxylase
ubiquinone biosynthesis monooxygenase Coq7
succinate dehydrogenase flavoprotein subunit
succinate dehydrogenase iron-sulfur protein
NADH dehydrogenase
NADH dehydrogenase
NADH dehydrogenase I subunit A
NADH dehydrogenase I subunit B
NADH dehydrogenase I subunit C
NADH dehydrogenase I subunit D
NADH dehydrogenase I subunit E
NADH dehydrogenase I subunit F
NADH dehydrogenase I subunit G
NADH dehydrogenase I subunit H
NADH dehydrogenase I subunit I
NADH dehydrogenase I subunit J
NADH dehydrogenase I subunit K
NADH dehydrogenase I subunit L
NADH dehydrogenase I subunit M
NADH dehydrogenase I subunit N
NADH dehydrogenase I subunit N
NADH dehydrogenase
NADH dehydrogenase
ubiquinol-cytochrome c reductase iron-sulfur subunit
ubiquinol-cytochrome c reductase cytochrome b subunit
ubiquinol-cytochrome c reductase cytochrome c1 subunit
inorganic pyrophosphatase
F-type H+-transporting ATPase subunit a
F-type H+-transporting ATPase subunit b
F-type H+-transporting ATPase subunit b
F-type H+-transporting ATPase subunit c
F-type H+-transporting ATPase subunit alpha
F-type H+-transporting ATPase subunit beta
F-type H+-transporting ATPase subunit delta
F-type H+-transporting ATPase subunit epsilon
F-type H+-transporting ATPase subunit gamma
cytochrome c oxidase subunit I
cytochrome c oxidase subunit II
cytochrome c oxidase subunit III
protoheme IX farnesyltransferase
F-type H+-transporting ATPase subunit a
F-type H+-transporting ATPase subunit b

F-type H+-transporting ATPase subunit b
F-type H+-transporting ATPase subunit c
F-type H+-transporting ATPase subunit alpha
F-type H+-transporting ATPase subunit beta
F-type H+-transporting ATPase subunit delta
F-type H+-transporting ATPase subunit epsilon
F-type H+-transporting ATPase subunit gamma
IMP dehydrogenase
ribonucleoside-diphosphate reductase alpha chain
ribonucleoside-diphosphate reductase beta chain
phosphoribosylaminoimidazolecarboxamide formyltransferase / IMP cyclohydrolase
amidophosphoribosyltransferase
adenylate kinase
nucleoside-diphosphate kinase
guanylate kinase
ribose-phosphate pyrophosphokinase
polyribonucleotide nucleotidyltransferase
dGTPase
5-(carboxyamino)imidazole ribonucleotide mutase
5-(carboxyamino)imidazole ribonucleotide synthase
adenylosuccinate lyase
phosphoribosylaminoimidazole-succinocarboxamide synthase
phosphoribosylformylglycinamidine cyclo-ligase
adenylosuccinate synthase
phosphoribosylamine--glycine ligase
GMP synthase (glutamine-hydrolysing)
phosphoribosylformylglycinamidine synthase
phosphoribosylformylglycinamidine synthase
DNA polymerase I
DNA polymerase III subunit alpha
DNA polymerase III subunit beta
DNA polymerase III subunit delta
DNA polymerase III subunit delta'
DNA polymerase III subunit epsilon
DNA polymerase III subunit gamma/tau
DNA-directed RNA polymerase subunit alpha
DNA-directed RNA polymerase subunit beta
DNA-directed RNA polymerase subunit beta'
DNA-directed RNA polymerase subunit omega
5'-nucleotidase
phosphoribosylglycinamide formyltransferase 1
dihydroorotate dehydrogenase (fumarate)
thioredoxin reductase (NADPH)
thioredoxin reductase (NADPH)
ribonucleoside-diphosphate reductase alpha chain
ribonucleoside-diphosphate reductase beta chain
aspartate carbamoyltransferase catalytic subunit

orotate phosphoribosyltransferase
nucleoside-diphosphate kinase
dTMP kinase
cytidylate kinase
polyribonucleotide nucleotidyltransferase
dihydroorotase
cytosine deaminase
dCTP deaminase
dUTP pyrophosphatase
orotidine-5'-phosphate decarboxylase
CTP synthase
carbamoyl-phosphate synthase large subunit
carbamoyl-phosphate synthase small subunit
DNA polymerase I
DNA polymerase III subunit alpha
DNA polymerase III subunit beta
DNA polymerase III subunit delta
DNA polymerase III subunit delta'
DNA polymerase III subunit epsilon
DNA polymerase III subunit gamma/tau
DNA-directed RNA polymerase subunit alpha
DNA-directed RNA polymerase subunit beta
DNA-directed RNA polymerase subunit beta'
DNA-directed RNA polymerase subunit omega
thymidylate synthase (FAD)
5'-nucleotidase
uridylate kinase
glutamate dehydrogenase
aspartate carbamoyltransferase catalytic subunit
amidophosphoribosyltransferase
aspartate aminotransferase
argininosuccinate lyase
adenylosuccinate lyase
glutamine synthetase
glutamine synthetase
adenylosuccinate synthase
argininosuccinate synthase
carbamoyl-phosphate synthase large subunit
carbamoyl-phosphate synthase small subunit
proline dehydrogenase / delta 1-pyrroline-5-carboxylate dehydrogenase
aspartate-semialdehyde dehydrogenase
dihydrolipoamide dehydrogenase
dihydrolipoamide dehydrogenase
glycine hydroxymethyltransferase
5-aminolevulinate synthase
aspartate kinase
phosphatidylserine synthase

aspartate-semialdehyde dehydrogenase
S-adenosylmethionine synthetase
aspartate aminotransferase
aspartate kinase
dihydrolipoamide dehydrogenase
dihydrolipoamide dehydrogenase
propionyl-CoA carboxylase alpha chain
propionyl-CoA carboxylase beta chain
pyruvate dehydrogenase E1 component subunit alpha
pyruvate dehydrogenase E1 component subunit beta
leucyl-tRNA synthetase
isoleucyl-tRNA synthetase
valyl-tRNA synthetase
aspartate-semialdehyde dehydrogenase
N-acetyl-gamma-glutamyl-phosphate/N-acetyl-gamma-aminoadipyl-phosphate reductase
dihydrodipicolinate reductase
2,3,4,5-tetrahydropyridine-2-carboxylate N-succinyltransferase
acetylornithine/N-succinyldiaminopimelate aminotransferase
aspartate kinase
acetylglutamate/acetylaminoadipate kinase
succinyl-diaminopimelate desuccinylase
diaminopimelate decarboxylase
dihydrodipicolinate synthase
diaminopimelate epimerase
2-oxoglutarate dehydrogenase E1 component
2-oxoglutarate dehydrogenase E2 component (dihydrolipoamide succinyltransferase)
--
N-acetyl-gamma-glutamyl-phosphate/N-acetyl-gamma-aminoadipyl-phosphate reductase
glutamate dehydrogenase
pyrroline-5-carboxylate reductase
ornithine carbamoyltransferase
glutamate N-acetyltransferase / amino-acid N-acetyltransferase
aspartate aminotransferase
acetylornithine/N-succinyldiaminopimelate aminotransferase
acetylglutamate/acetylaminoadipate kinase
cytosine deaminase
argininosuccinate lyase
glutamine synthetase
glutamine synthetase
argininosuccinate synthase
proline dehydrogenase / delta 1-pyrroline-5-carboxylate dehydrogenase
--
--
--
--
aspartate aminotransferase
aspartate aminotransferase

--
protocatechuate 3,4-dioxygenase, beta subunit
--
--
--
2-oxoglutarate dehydrogenase E1 component
tryptophanyl-tRNA synthetase
3-phosphoshikimate 1-carboxyvinyltransferase
aspartate aminotransferase
aspartate aminotransferase
malonyl-CoA decarboxylase
thioredoxin reductase (NADPH)
thioredoxin reductase (NADPH)
methionyl-tRNA synthetase
glycine hydroxymethyltransferase
isocitrate dehydrogenase
glutathione S-transferase
leucyl aminopeptidase
glutamate--cysteine ligase
glutathione synthase
glucose-1-phosphate adenylyltransferase
glucose-1-phosphate adenylyltransferase
myo-inositol-1(or 4)-monophosphatase
D-alanyl-D-alanine carboxypeptidase (penicillin-binding protein 5/6)
1-acyl-sn-glycerol-3-phosphate acyltransferase
glycerol-3-phosphate acyltransferase PlsX
glycerol-3-phosphate acyltransferase PlsY
myo-inositol-1(or 4)-monophosphatase
triosephosphate isomerase (TIM)
glycerol-3-phosphate dehydrogenase (NAD(P)+)
1-acyl-sn-glycerol-3-phosphate acyltransferase
phosphatidate cytidylyltransferase
CDP-diacylglycerol--glycerol-3-phosphate 3-phosphatidyltransferase
phosphatidylserine synthase
phosphatidylglycerophosphatase A
phosphatidylserine decarboxylase
phosphatidylserine decarboxylase
glycerol-3-phosphate acyltransferase PlsX
glycerol-3-phosphate acyltransferase PlsY
--
malate dehydrogenase
malate dehydrogenase (oxaloacetate-decarboxylating)(NADP+)
pyruvate dehydrogenase E1 component subunit alpha
pyruvate dehydrogenase E1 component subunit beta
dihydrolipoamide dehydrogenase
dihydrolipoamide dehydrogenase
pyruvate dehydrogenase E2 component (dihydrolipoamide acetyltransferase)

pyruvate,orthophosphate dikinase
succinate dehydrogenase flavoprotein subunit
succinate dehydrogenase iron-sulfur protein
--
protocatechuate 3,4-dioxygenase, beta subunit
--
--
--
--
--
--
--
malate dehydrogenase
phosphoglycolate phosphatase
citrate synthase
aconitate hydratase 1
propionyl-CoA carboxylase alpha chain
propionyl-CoA carboxylase beta chain
malonyl-CoA decarboxylase
succinyl-CoA synthetase alpha subunit
succinyl-CoA synthetase beta subunit
propionyl-CoA carboxylase alpha chain
propionyl-CoA carboxylase beta chain
--
--
pyruvate dehydrogenase E1 component subunit alpha
pyruvate dehydrogenase E1 component subunit beta
succinate dehydrogenase flavoprotein subunit
succinate dehydrogenase iron-sulfur protein
succinyl-CoA synthetase alpha subunit
succinyl-CoA synthetase beta subunit
glycine hydroxymethyltransferase
phosphoribosylaminoimidazolecarboxamide formyltransferase / IMP cyclohydrolase
methionyl-tRNA formyltransferase
methylenetetrahydrofolate dehydrogenase (NADP+) / methenyltetrahydrofolate cyclohydrolase
5-formyltetrahydrofolate cyclo-ligase
thymidylate synthase (FAD)
phosphoribosylglycinamide formyltransferase 1
malate dehydrogenase
glycine hydroxymethyltransferase
enolase
phosphoglycerate mutase
malate dehydrogenase
malate dehydrogenase (oxaloacetate-decarboxylating)(NADP+)
transketolase
aspartate aminotransferase
phosphoglycerate kinase

pyruvate,orthophosphate dikinase
fructose-bisphosphate aldolase, class I
ribulose-phosphate 3-epimerase
triosephosphate isomerase (TIM)
ribose 5-phosphate isomerase B
fructose-1,6-bisphosphatase II
malate dehydrogenase
isocitrate dehydrogenase
succinate dehydrogenase flavoprotein subunit
succinate dehydrogenase iron-sulfur protein
methylenetetrahydrofolate dehydrogenase (NADP+) / methenyltetrahydrofolate cyclohydrolase
fumarate hydratase, class II
aconitate hydratase 1
succinyl-CoA synthetase alpha subunit
succinyl-CoA synthetase beta subunit
propionyl-CoA carboxylase alpha chain
propionyl-CoA carboxylase beta chain
thiamine-phosphate pyrophosphorylase
hydroxymethylpyrimidine/phosphomethylpyrimidine kinase
thiamine-monophosphate kinase
adenylyltransferase
glycine oxidase
cysteine desulfurase
cysteine desulfurase
riboflavin synthase
6,7-dimethyl-8-ribityllumazine synthase
GTP cyclohydrolase II
3,4-dihydroxy 2-butanone 4-phosphate synthase
diaminohydroxyphosphoribosylaminopyrimidine deaminase / 5-amino-6-(5-phosphoribosylamino)uracil reductase
riboflavin kinase / FMN adenylyltransferase
pyridoxamine 5'-phosphate oxidase
pyridoxine 5-phosphate synthase
nicotinate-nucleotide pyrophosphorylase (carboxylating)
NAD+ kinase
nicotinate-nucleotide adenylyltransferase
NAD+ synthase (glutamine-hydrolysing)
quinolinate synthase
5'-nucleotidase
dephospho-CoA kinase
pantetheine-phosphate adenylyltransferase
holo-[acyl-carrier protein] synthase
phosphopantothenoylcysteine decarboxylase
phosphopantothenate-cysteine ligase
type III pantothenate kinase
8-amino-7-oxononanoate synthase
adenosylmethionine-8-amino-7-oxononanoate aminotransferase
biotin synthetase

--
dethiobiotin synthetase
BirA family transcriptional regulator, biotin operon repressor / biotin-[acetyl-CoA-carboxylase] ligase
lipoic acid synthetase
lipoyl(octanoyl) transferase
dihydropteroate synthase
2-amino-4-hydroxy-6-hydroxymethyldihydropteridine diphosphokinase
GTP cyclohydrolase I
dihydroneopterin aldolase
dihydrofolate synthase / folylpolyglutamate synthase
coproporphyrinogen III oxidase
5-aminolevulinate synthase
uroporphyrinogen decarboxylase
porphobilinogen synthase
uroporphyrinogen-III synthase
hydroxymethylbilane synthase
ferrochelatase
glutamyl-tRNA synthetase
glutamyl-tRNA synthetase
protoheme IX farnesyltransferase
1-deoxy-D-xylulose-5-phosphate reductoisomerase
farnesyl diphosphate synthase
4-diphosphocytidyl-2-C-methyl-D-erythritol kinase
2-C-methyl-D-erythritol 4-phosphate cytidylyltransferase
2-C-methyl-D-erythritol 2,4-cyclodiphosphate synthase
octaprenyl-diphosphate synthase
(E)-4-hydroxy-3-methylbut-2-enyl-diphosphate synthase
4-hydroxy-3-methylbut-2-enyl diphosphate reductase
--
--
tRNA dimethylallyltransferase
glutamate dehydrogenase
glutamine synthetase
glutamine synthetase
aspartate aminotransferase
aspartate aminotransferase
methionyl-tRNA formyltransferase
tyrosyl-tRNA synthetase
tryptophanyl-tRNA synthetase
threonyl-tRNA synthetase
leucyl-tRNA synthetase
isoleucyl-tRNA synthetase
alanyl-tRNA synthetase
valyl-tRNA synthetase
methionyl-tRNA synthetase
seryl-tRNA synthetase
aspartyl-tRNA synthetase

glycyl-tRNA synthetase alpha chain
glycyl-tRNA synthetase beta chain
prolyl-tRNA synthetase
cysteinyl-tRNA synthetase
glutamyl-tRNA synthetase
glutamyl-tRNA synthetase
arginyl-tRNA synthetase
phenylalanyl-tRNA synthetase alpha chain
phenylalanyl-tRNA synthetase beta chain
histidyl-tRNA synthetase
aspartyl-tRNA(Asn)/glutamyl-tRNA (Gln) amidotransferase subunit A
aspartyl-tRNA(Asn)/glutamyl-tRNA (Gln) amidotransferase subunit B
aspartyl-tRNA(Asn)/glutamyl-tRNA (Gln) amidotransferase subunit C
lysyl-tRNA synthetase, class I
glutathione S-transferase
glutathione S-transferase
IMP dehydrogenase
GMP synthase (glutamine-hydrolysing)
3-oxoacyl-[acyl-carrier protein] reductase
transketolase
iron(III) transport system ATP-binding protein
phosphate transport system ATP-binding protein
heme exporter protein A
lipoprotein-releasing system ATP-binding protein
zinc transport system ATP-binding protein
glutamine synthetase
glutamine synthetase
two-component system, cell cycle sensor histidine kinase PleC
two-component system, NtrC family, nitrogen regulation sensor histidine kinase NtrY
ribonuclease III
polyribonucleotide nucleotidyltransferase
poly(A) polymerase
enolase
ribonuclease E
DNA-directed RNA polymerase subunit alpha
DNA-directed RNA polymerase subunit beta
DNA-directed RNA polymerase subunit beta'
DNA-directed RNA polymerase subunit omega
DNA ligase (NAD+)
replicative DNA helicase
DNA primase
DNA polymerase I
DNA polymerase III subunit alpha
DNA polymerase III subunit beta
DNA polymerase III subunit delta
DNA polymerase III subunit delta'
DNA polymerase III subunit epsilon

DNA polymerase III subunit gamma/tau
ribonuclease HI
ribonuclease HII
signal peptidase I
signal peptidase II
exodeoxyribonuclease III
DNA ligase (NAD+)
DNA polymerase I
DNA-3-methyladenine glycosylase
single-stranded-DNA-specific exonuclease
endonuclease III
DNA ligase (NAD+)
DNA polymerase I
DNA helicase II / ATP-dependent DNA helicase PcrA
transcription-repair coupling factor (superfamily II helicase)
DNA ligase (NAD+)
DNA polymerase III subunit alpha
DNA polymerase III subunit beta
DNA polymerase III subunit delta
DNA polymerase III subunit delta'
DNA polymerase III subunit epsilon
DNA polymerase III subunit gamma/tau
exodeoxyribonuclease VII large subunit
exodeoxyribonuclease VII small subunit
DNA helicase II / ATP-dependent DNA helicase PcrA
single-stranded-DNA-specific exonuclease
crossover junction endodeoxyribonuclease RuvC
DNA polymerase I
DNA polymerase III subunit alpha
DNA polymerase III subunit beta
DNA polymerase III subunit delta
DNA polymerase III subunit delta'
DNA polymerase III subunit epsilon
DNA polymerase III subunit gamma/tau
ATP-dependent DNA helicase RecG
primosomal protein N' (replication factor Y) (superfamily II helicase)
single-stranded-DNA-specific exonuclease
phosphatidate cytidylyltransferase
myo-inositol-1(or 4)-monophosphatase
ATP-dependent Lon protease
ATP-dependent Clp protease, protease subunit
replicative DNA helicase
two-component system, cell cycle sensor histidine kinase PleC
regulator of sigma E protease
tRNA-specific 2-thiouridylase
adenylyltransferase
cysteine desulfurase

cysteine desulfurase
isocitrate dehydrogenase
malonyl-CoA decarboxylase
superoxide dismutase, Fe-Mn family
ubiquinol-cytochrome c reductase iron-sulfur subunit
ubiquinol-cytochrome c reductase cytochrome b subunit
ubiquinol-cytochrome c reductase cytochrome c1 subunit
glutamine synthetase
glutamine synthetase
glyceraldehyde 3-phosphate dehydrogenase
ubiquinol-cytochrome c reductase iron-sulfur subunit
ubiquinol-cytochrome c reductase cytochrome b subunit
ubiquinol-cytochrome c reductase cytochrome c1 subunit
ubiquinol-cytochrome c reductase iron-sulfur subunit
ubiquinol-cytochrome c reductase cytochrome b subunit
ubiquinol-cytochrome c reductase cytochrome c1 subunit
ubiquinol-cytochrome c reductase iron-sulfur subunit
ubiquinol-cytochrome c reductase cytochrome b subunit
ubiquinol-cytochrome c reductase cytochrome c1 subunit
superoxide dismutase, Fe-Mn family
fumarate hydratase, class II
