# Supplementary material for: Whole Genome Sequencing and Comparative Analysis of the First Ehrlichia canis Isolate in China
Source: Microorganisms. 2024 Jan 8;12(1):125. doi: 10.3390/microorganisms12010125 (PMC10820421; doi:10.3390/microorganisms12010125)
Supplement: Supplementary file 1 [file microorganisms-12-00125-s001.zip › microorganisms-2797756-supplementary.pdf]

microorganisms

Supplementary Table S1. Pathogen-host interactions analysis of *E. canis* YZ-1

Gene-ID	Identity	Gene-Name	Protein-Accession	Species	Phenotype
GM000005	60.0	*dnaJ*	CAG46145	*Francisella tularensis*	unaffected pathogenicity
GM000101	50.0	*smpB*	SSRP_SALTY	*Salmonella enterica*	reduced virulence
GM000119	44.3	*XAC1722*	AAM36589	*Xanthomonas citri*	reduced virulence
GM000135	40.0	*MacB*	AAL19878	*Salmonella enterica*	reduced virulence
GM000144	49.2	*nrdR*	AAK33390	*Streptococcus pyogenes*	reduced virulence
GM000176	40.3	*thioredoxin 1*	BAA00903	*Salmonella enterica*	reduced virulence
GM000185	50.0	*IMPDH*	ADE32434	*Streptococcus suis*	reduced virulence
GM000272	43.1	*GzOB048*	FGSG_13818	*Fusarium graminearum*	unaffected pathogenicity
GM000287	51.9	*glyA*	ACR70340	*Edwardsiella ictaluri*	reduced virulence
GM000289	48.0	*MacB*	AAL19878	*Salmonella enterica*	reduced virulence
GM000291	59.2	*frdA*	ACR67633	*Edwardsiella ictaluri*	reduced virulence
GM000333	52.2	*GroEL*	BAA04222	*Porphyromonas gingivalis*	increased virulence (hypervirulence)
GM000385	40.4	*secY*	CAD00690	*Listeria monocytogenes*	increased virulence (hypervirulence)
GM000400	52.4	*pstB*	AAM36442	*Xanthomonas citri*	loss of pathogenicity
GM000415	57.9	*purA*	AAL23186	*Salmonella enterica*	reduced virulence
GM000424	40.7	*RNase E*	ADX16885	*Salmonella enterica*	reduced virulence
GM000469	45.8	*znuB*	ACA68428	*Yersinia pseudotuberculosis*	reduced virulence
GM000472	63.3	*ubiquinol oxidase*	AAK26622	*Blumeria graminis*	chemistry target: resistance to chemical
GM000521	46.2	*PSPTO_2691*	AAO56193	*Pseudomonas syringae*	unaffected pathogenicity
GM000622	47.4	*MoARG1*	EHA55560	*Magnaporthe oryzae*	loss of pathogenicity
GM000653	47.5	*pnp*	AE004888_1	*Pseudomonas aeruginosa*	reduced virulence
GM000659	53.8	*Bx-Prx*	ABW81468	*Bursaphelenchus xylophilus*	loss of pathogenicity
GM000660	70.9	*TRR1*	AY796185	*Cryptococcus neoformans*	Lethal

GM0006 91	41.7	*CovR*	ACE75886	*Streptococcus pyogenes*	increased virulence (hypervirulence)
GM0007 32	47.4	*SSBX*	AAW74693	*Xanthomonas oryzae*	reduced virulence
GM0007 65	60.0	*HSP90*	AAA02743	*Saccharomyces cerevisiae*	increased virulence (hypervirulence)
GM0007 68	59.6	*GyrA*	BAD26622	*Flavobacterium psychrophilum*	chemistry target: sensitivity to chemical
Continu ed					
GM0007 92	43.2	*argD*	CBA23647	*Erwinia amylovora*	reduced virulence
GM0008 04	55.6	*LonA*	ABN73480	*Actinobacillus pleuropneumoniae*	reduced virulence
GM0008 05	53.3	*ClpX*	BAF67840	*Staphylococcus aureus*	reduced virulence
GM0008 06	76.6	*ClpP*	BAA94668	*Salmonella enterica*	reduced virulence
GM0008 29	48.3	*argH*	ABD20820.1	*Staphylococcus aureus*	reduced virulence
GM0008 55	57.1	*LssD*	CAD90950	*Legionella pneumophila*	reduced virulence
GM0009 03	40.4	*ADE2*	CAA99327	*Saccharomyces cerevisiae*	reduced virulence
GM0009 21	41.0	*RNase III*	AAA92440	*Salmonella enterica*	reduced virulence
GM0009 63	49.0	*recA*	AAL00560	*Streptococcus pneumoniae*	reduced virulence
GM0009 69	51.7	*XC_2203*	AAY49259	*Xanthomonas campestris*	reduced virulence
GM0010 22	56.1	*MGG_00383*	MGG_00383	*Magnaporthe oryzae*	reduced virulence

Supplemental Table S2. All the genes of *E. canis* YZ-1 identified in KEGG and TC system analysis

Please find the additional PDF file in the supplementary. Thanks!

Supplemental Table S3. SNPs (single nucleotide polymorphisms) analysis of *E. canis* YZ-1 and related *Ehrlichia* stains

Reference	GenBank #	St-s*	Sp-s	St-ns	Sp-ns	Pm-sp	Syn	N-syn	T-CDS SNP	In-genic	T-SNP
E. chaffeensis	CP000236	6	49	25	20	215	51,041	26,881	78,152	4,914	83,066
Ehrlichia sp.	NZ_CP007474	3	43	17	19	168	48,771	26,829	75,818	5,198	81,016
E. muris	CP006917	4	47	19	22	165	46,912	25,518	72,655	6,922	79,577
E. ruminantium	CR767821	1	18	10	6	142	21,627	11,399	33,192	1,566	34,758
E. mineirensis	NZ_CDGH01000070	3	88	19	38	174	34,778	22,417	57,502	23,823	81,325
E. canis	NC_007354	0	0	1	2	13	328	1,090	1,433	315	1,748

*St-s: start-codon synonymous SNPs; Sp-s: stop-codon synonymous SNPs; St-ns, start-codon nonsynonymous SNPs; Sp-ns: stop-codon nonsynonymous SNPs; Pm-sp: premature stop-codon mutation; Syn: synonymous mutation inside the gene; N-syn: nonsynonymous mutations inside the gene; T-CDS SNP: total coding sequencing (CDS) SNP; In-genic: intergenic SNPs; T-SNP: total SNP.

Supplemental Table S4. InDel (insertions and deletions) analysis of *E. canis* YZ-1 and related *Ehrlichia* stains

Reference	GenBank #	St-co ins*	CDS inside ins	Sp-co ins	St-co del	CDS inside del	Sp-co del
E. chaffeensis	CP000236	1	13	2	1	20	0
Ehrlichia sp.	NZ_CP007474	1	6	0	0	14	0
E. muris	CP006917	1	5	0	0	9	0
E. ruminantium	CR767821	0	7	0	0	5	1
E. mineirensis	NZ_CDGH01000070	1	9	0	1	9	0
E.canis	NC_007354	0	17	0	0	16	0

*St-co ins: start-codon insertions; CDS inside ins: coding sequencing-inside insertion; Sp-co ins: stop-codon insertion; St-co del: start-codon deletion; CDS inside del: CDS-inside deletion; Sp-co del: stop-codon deletion.

Supplemental Table S5. Mutation type caused by InDel (insertions and deletions) in *E. canis* YZ-1 and related *Ehrlichia* stains

Reference	GenBank #	Frame-shifted*	St-co	Sp-co	Premature sp	Effect	CDS with indel	All CDS
E. chaffeensis	CP000236	13	0	0	0	24	37	1105
Ehrlichia sp.	NZ_CP007474	7	0	0	0	13	20	855
E. muris	CP006917	4	0	0	0	11	15	904
E. ruminantium	CR767821	4	0	0	1	8	13	888
E. mineirensis	NZ_CDGH01000070	5	0	1	2	10	18	933
E. canis	NC_007354	15	0	0	3	7	25	925

*Frame-shifted: frameshift mutations; St-co: start codon; Sp-co: stop codon; Premature sp: premature stop; Effect: no effects on open reading frame; CDS with indel: number of CDS with insertions or deletions.

Supplemental Table S6. The SV types of *E. canis* YZ-1 and related *Ehrlichia* stains

Reference	GenBank #	SV type*								
		Tr	Iv	Tr+Iv	De	Is	CID	DI	DD	Total
E. chaffeensis	CP000236	7	7	7	49	80	184	0	0	334
Ehrlichia sp.	NZ_CP007474	7	8	97	44	77	160	0	0	393
E. muris	CP006917	10	14	94	47	71	166	0	0	402
E. ruminantium	CR767821	19	9	130	98	21	248	0	0	525
E. mineirensis	NZ_CDGH01000070	43	25	1	28	46	4	0	0	147
E. canis	NC_007354	1	10	4	13	13	1	0	0	42

*Tr: translocation; Iv: inversion; Tr+Iv: Translocation + inversion; De: deletion; Is: insertion; CID: complex InDel, (The sequence corresponding to the position of the reference genome and the sample genome cannot be aligned. The reason may be that both the reference sequence and the studied sequence have been inserted in the specified region, or that one of them has been inserted or deleted in the overlapped region, or the variation is too large to be compared); DI: Duplicate Insertion; DD: Duplicate Deletion.

Figure S1

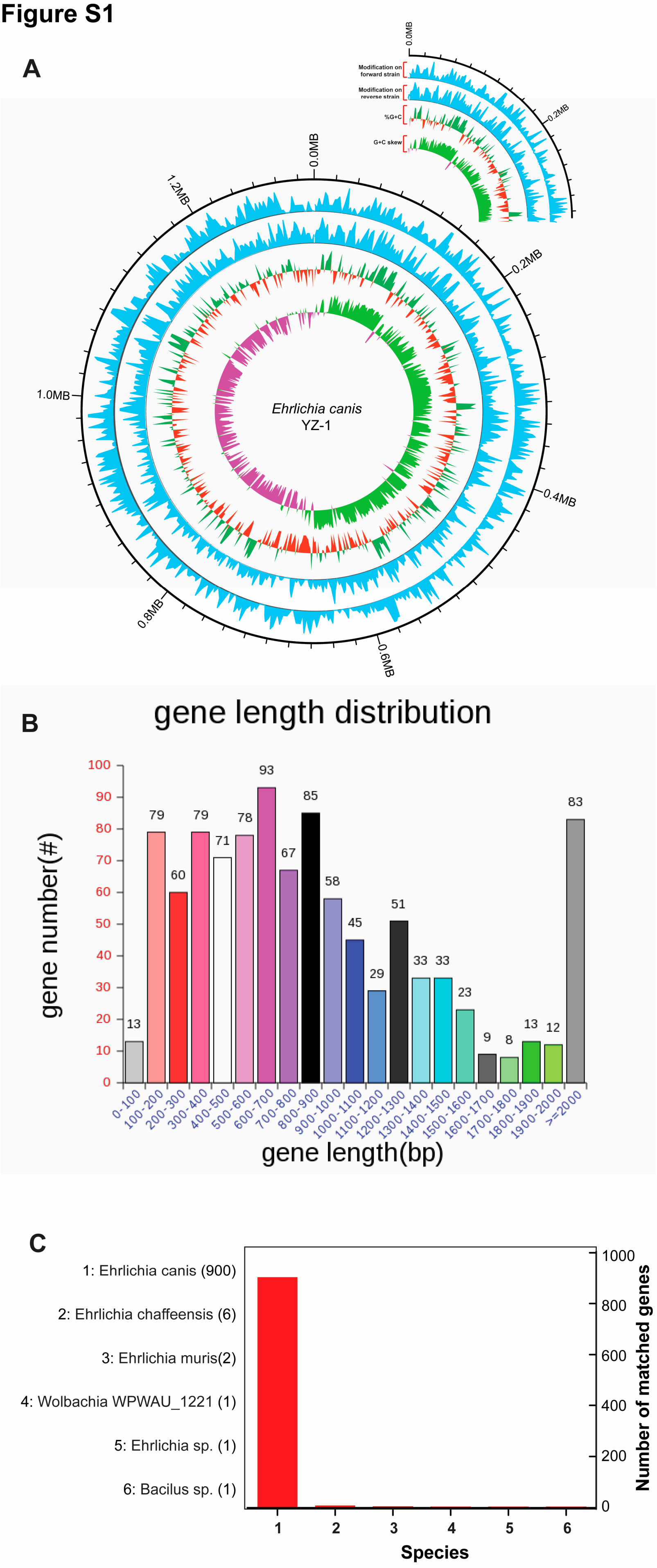
A
0.0MB
0.2MB
0.4MB
0.6MB
0.8MB
1.0MB
1.2MB
Ehrlichia canis
YZ-1
Modification on forward strain
Modification on reverse strain
%G+C
G+C skew
B
gene length distribution
gene number(#)
100
90
80
70
60
50
40
30
20
10
0
13
79
60
79
71
78
93
67
85
58
45
29
51
33
33
23
9
8
13
12
83
0-100
100-200
200-300
300-400
400-500
500-600
600-700
700-800
800-900
900-1000
1000-1100
1100-1200
1200-1300
1300-1400
1400-1500
1500-1600
1600-1700
1700-1800
1800-1900
1900-2000
>=2000
gene length(bp)
C
1: Ehrlichia canis (900)
2: Ehrlichia chaffeensis (6)
3: Ehrlichia muris(2)
4: Wolbachia WPWAU_1221 (1)
5: Ehrlichia sp. (1)
6: Bacilus sp. (1)
1000
800
600
400
200
0
Number of matched genes
1
2
3
4
5
6
Species

Figure S1. The epigenetic modifications of *Ehrlichia canis* YZ-1. (**A**) As indicated in the upright corner of the figure, the outermost track and the next track show the epigenetic modifications in the forward and reverse direction, together with the GC content in the next track and GC skew values in the inner tack. (**B**) The predicted gene distribution of *E. canis* YZ-1. The number (Y-axis) and length in base-pair (bp) (X-axis) of the genes identified in *E. canis* YZ-1 were colored here. (**C**) The top 6 species matched with the *E. canis* YZ-1 in this study when BLASTed using the NCBI NR database.

Figure S2

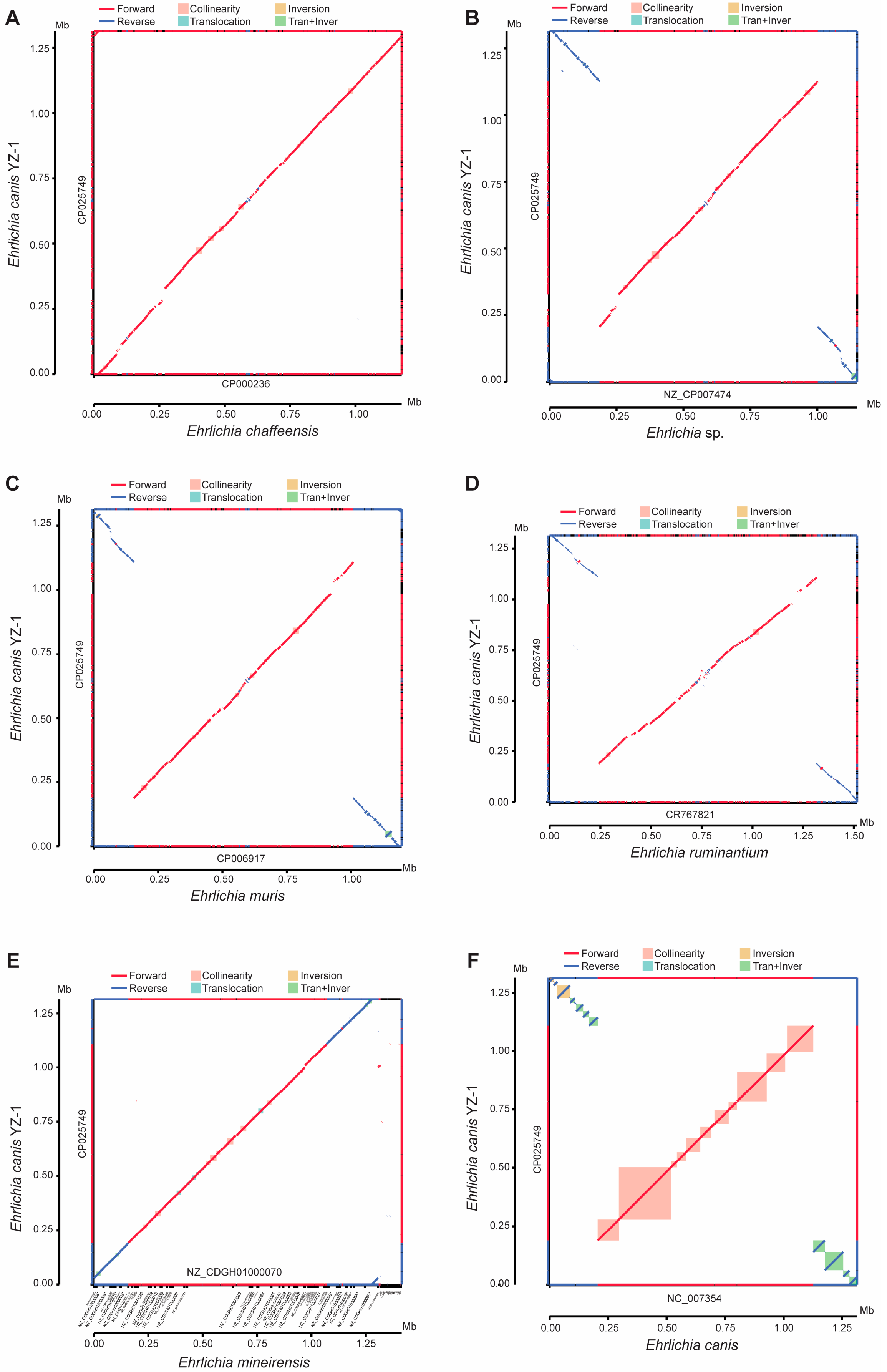
A
Forward
Reverse
Collinearity
Translocation
Inversion
Tran+Inver
Mb
CP025749
CP000236
Ehrlichia canis YZ-1
Ehrlichia chaffeensis
B
NZ_CP007474
Ehrlichia sp.
C
CP006917
Ehrlichia muris
D
CR767821
Ehrlichia ruminantium
E
NZ_CDGH01000070
Ehrlichia mineirensis
F
NC_007354
Ehrlichia canis
0.00
0.25
0.50
0.75
1.00
1.25
1.50

Figure S2. Bidimensional synteny analysis of *Ehrlichia canis* YZ-1 with the reference sequences. The bidimensional synteny analysis was performed on E. canis YZ1 from our study and *E. chaffeensis* (GenBank accession number CP000236) (**A**), *Ehrlichia* sp. (NZ_CP007474) (**B**), *E. muris* (CP006917) (**C**), *E. ruminantium* (CR767821) (**D**), *E. mineirensis* (NZ_CDGH01000070) (**E**), and *E. canis* (NC_007354) (**F**) from NCBI, with collinearity (pink), inversion (orange), translocation (azure), and translocation + inversion (green).

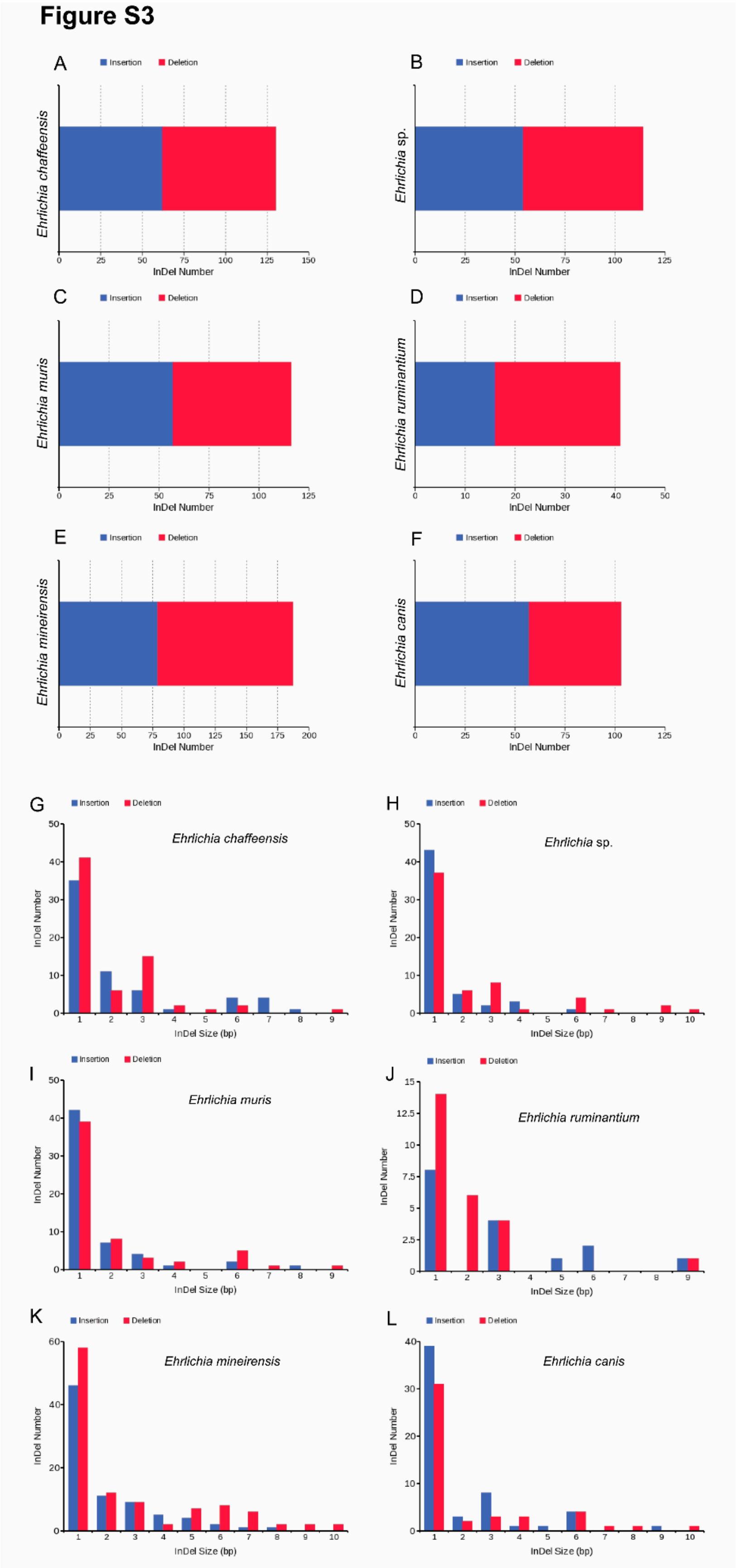

Figure S3. The comparative analysis of InDels between *Ehrlichia canis* YZ-1 and reference sequences. The number (**A-F**) and length (**G-L**) of insertion (blue) and deletion (red) mutations were aligned between *E. canis* YZ-1 from this

study and Ehrlichia references published before, including *E. chaffeensis* (GenBank accession number .CP000236) (**A and G**), *Ehrlichia* sp. (NZ_CP007474) (**B and H**), *E. muris* (CP006917) (**C and I**), *E. ruminantium* (CR767821) (**D and J**), *E. mineirensis* (NZ_CDGH01000070) (**E and K**), and *E. canis* (NC_007354) (**F and L**).

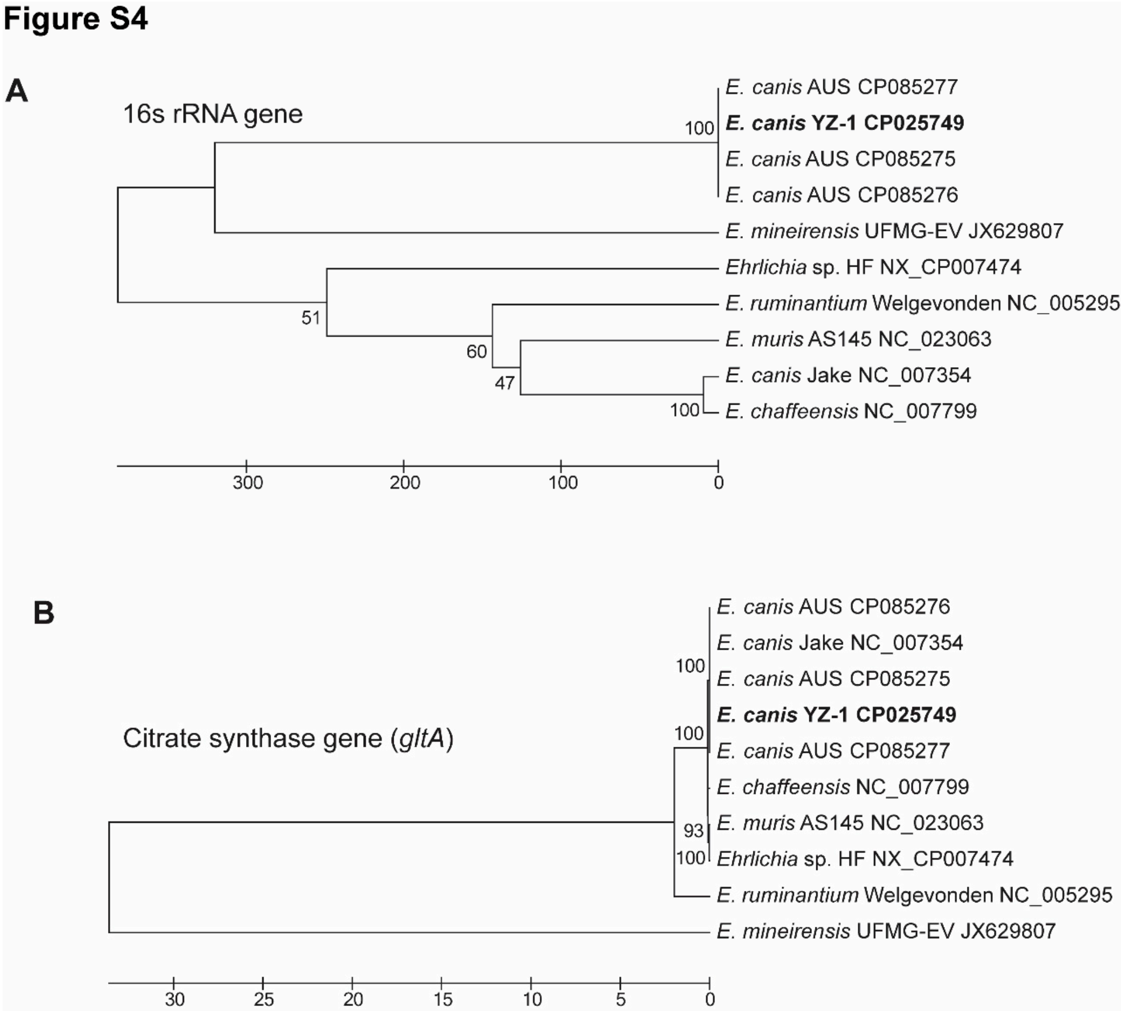

Figure S4. Phylogenetic relationships of *Ehrlichia canis* YZ-1 and references. The phylogenetic tree of Ehrlichia canis YZ-1 and related references was interred on 16s rRNA gene (**A**) and Citrate synthase gene (*gltA*) (**B**) using the UPGMA method and conducted in MEGA6. The percentage of replicate trees in which the associated taxa clustered together in the bootstrap test (500 replicates) are shown next to the branches. The tree is drawn to scale, with branch lengths in the same units as those of the evolutionary distances used to infer the phylogenetic tree. The evolutionary distances were computed using the Maximum Composite Likelihood method and are in the units of the number of base substitutions per site. The rate variation among sites was modeled with a gamma distribution (shape parameter = 1).
